# Persistence of High Systemic Immune-Inflammation Index as a Predictor of In-Hospital Mortality in COVID-19 Patients

**DOI:** 10.7759/cureus.90333

**Published:** 2025-08-17

**Authors:** Anggia F Agustin, Rizka Humardewayanti Asdie, Deshinta Putri Mulya

**Affiliations:** 1 Department of Internal Medicine, Dr. Sardjito General Hospital, Yogyakarta, IDN; 2 Faculty of Medicine, Public Health, and Nursing, Universitas Gadjah Mada, Yogyakarta, IDN; 3 Division of Tropical Infection, Department of Internal Medicine, Dr. Sardjito General Hospital, Yogyakarta, IDN; 4 Division of Allergy and Immunology, Department of Internal Medicine, Dr. Sardjito General Hospital, Yogyakarta, IDN

**Keywords:** covid-19, high persistence, in-hospital mortality, sars-cov-2, systemic immune-inflammation index

## Abstract

Background: The SARS-CoV-2 infection causes an exaggeration in immune cell activity and massive production of cytokines and inflammatory mediators. This is accompanied by severe lymphopenia, thrombosis, and multiorgan mononuclear infiltration, which determine the severity and mortality of COVID-19. The systemic immune-inflammation index (SII), based on the number of neutrophils, lymphocytes, and platelets, reflects ongoing inflammation and immune status. This study aims to assess the value of the persistence of a high SII in predicting in-hospital mortality of COVID-19 patients.

Methods: A retrospective cohort observational study was conducted using data from COVID-19 patients between January and December 2021. The high SII cut-off value was determined using receiver operating characteristic (ROC) analysis. Bivariate analysis was used to determine the relationship between in-hospital mortality and the persistence of high SII and other variables, followed by a multivariate logistic regression model analysis to determine the clinical features associated with in-hospital COVID-19 mortality.

Results: There were 310 COVID-19 patients enrolled, with a mortality rate of 30%. The SII cut-off value was 1942.5x10^9^, and the persistence of high SII was associated with hospital mortality. Multivariate analysis revealed that the administration of additional COVID-19 therapies, disease severity, persistence of high SII, and chronic kidney disease were independently associated with in-hospital mortality.

Conclusion: This study represents a significant association between the persistence of high SII as a prognostic factor for COVID-19 mortality.

## Introduction

COVID-19 is a disease caused by a beta coronavirus, severe acute respiratory syndrome coronavirus 2 (SARS‑CoV‑2) [1,2]. As of December 2021, Indonesia had recorded 4,259,644 positive cases of COVID-19 with a mortality rate of 3.3% [3]. The causes of death include acute respiratory distress syndrome (ARDS), sepsis, and septic shock with multi-organ failure. The average time from the first onset of symptoms to death ranges from 14 to 22 days. In 2020, the mortality rate in severe and critical cases of COVID-19 was as high as 38%, with a median length of stay of seven days in the intensive care unit (ICU), which occurred in Wuhan. This is exacerbated in patients of advanced age and with comorbidities such as hypertension, diabetes mellitus, obesity, heart disease, and chronic obstructive pulmonary disease (COPD) [4,5]. SARS-CoV-2 infection is thought to trigger an immune response, namely a cytokine storm characterized by increased activity of immune cells, massive production of cytokines, and inflammatory mediators [6].

The systemic immune-inflammation index (SII) is a parameter based on the number of neutrophils, lymphocytes, and platelets; these parameters are indicators of inflammation and can reflect immune status. The depiction of these parameter results that illustrate inflammation at high SII values includes severe lymphopenia, thrombosis, and infiltration of mononuclear cells in various organs. SII value has been used for inflammatory markers in malignancy and systemic conditions. It has a positive correlation with other ratios between C-reactive protein, albumin, neutrophil count, and lymphocyte count, SII value as an independent marker for inflammation activity [6,7]. The previous retrospective study demonstrated that SII can predict survival in COVID-19 patients, length of stay, and the likelihood of needing intensive care with a respirator compared to other inflammatory indices derived from routine blood tests [8-10]. However, research on the persistence of high SII values ​​in COVID-19 patients has not been studied before, especially in Indonesia. This study aims to determine whether persistent high SII is independently associated with in-hospital mortality among COVID-19 patients in Indonesia.

## Materials and methods

This was a retrospective cohort study conducted in the adult isolation unit of Dr. Sardjito General Hospital, Yogyakarta, Indonesia, between January and December 2021. The study was approved by the Medical and Health Research Ethics Committee of the Faculty of Medicine, Public Health, and Nursing, Universitas Gadjah Mada (approval number: KE/FK/0091/EC/2022). The study has also received permission from the Director of Dr. Sardjito General Hospital.

Eligibility criteria

The inclusion criteria were diagnosis of COVID-19 through an oropharyngeal and nasopharyngeal RT-PCR SARS-CoV-2 swab test, age 18 years or older, and hospitalization in the adult isolation unit for more than three days. Patients with comorbidities such as diabetes mellitus, hypertension, and chronic kidney disease were also included. Exclusion criteria were pregnancy, HIV infection, malignancy, chronic liver disease, tuberculosis or another chronic lung disease, having an autoimmune condition, or having a history of immunosuppressant therapy within the last month. If one of the exclusion criteria was confirmed, then the patient was excluded.

Data collection

Clinical and laboratory data of COVID-19 patients who met the eligibility criteria and other examinations as the patient's standard of care were collected from the patients' medical records. The initial SII value (results of the platelet count multiplied by the neutrophil count, divided by the lymphocyte count) was obtained during COVID-19 diagnosis. In contrast, the second SII value was derived from peripheral blood count taken on days 3-7 of treatment. Patients with persistently high SII values were then followed to find out the occurrence of death within the first 14 days of confirmed COVID-19 diagnosis. Sampling was conducted using consecutive sampling methods. The COVID-19 diagnosis was classified into two degrees of severity: (i) mild to moderate, meaning patients with clinical signs of pneumonia (fever, cough, shortness of breath, rapid breathing) but not severe pneumonia, including SpO2 ≥ 93% room air, and (ii) severe, meaning patients with clinical signs of pneumonia plus one of the following: respiratory rate > 30 breaths/minute, severe respiratory distress, or SpO2 < 93% on room air.

Data analysis

The optimal SII cut-off value for predicting COVID-19 mortality was determined by receiver operating characteristic (ROC) curve analysis with a 95% confidence interval (CI). In analytical statistics, bivariate analysis using the chi-square test compared the variables, and multivariate analysis was used to determine variables that serve as predictors for COVID-19 mortality. IBM SPSS Statistics for Windows, version 24 (IBM corp., Armonk, New York, United States) was used for statistical analysis.

## Results

**Figure 1:**
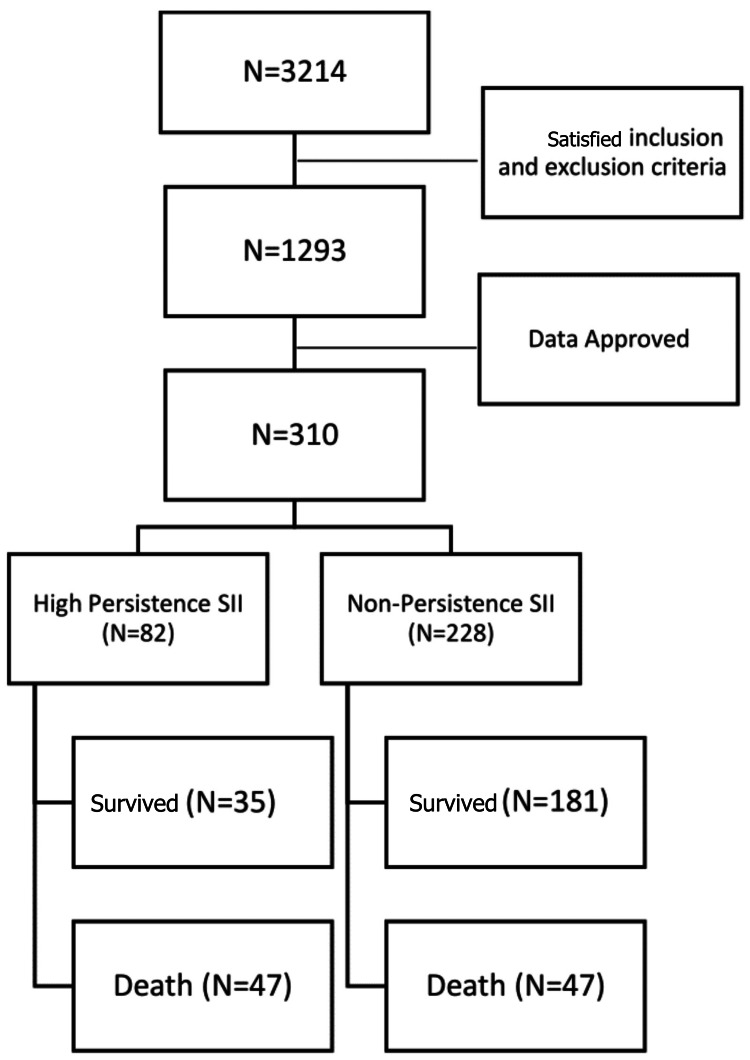
CONSORT flow chart; subject selection was based on criteria and data approved, and then divided into two groups (high persistence SII and non-persistence SII). N: Number of patients CONSORT: Consolidated Standards of Reporting Trials; SII: systemic immune-inflammation index

The study flow chart is illustrated in Figure 1. There were 310 subjects, of which 94 patients died (30.3%) and 216 survived (69.7%). The characteristics of the study subjects are shown in Table 1. Among the 310 subjects, 179 patients (57.7%) were male and 131 patients (42.3%) were female, with a median age of 57 years. At admission, the median platelet count was 211.5 × x10^3^/µL, the absolute neutrophil count was 5890x10^3^/µL, and the absolute lymphocyte count was 995x10^3^/µL with a median SII value of 1165.11x103/µL. After the first week of treatment, the median platelet count was 289.5 × x10^3^/µL (±136.5), the absolute neutrophil count was 8045x10^3^/µL, and the absolute lymphocyte count was 995x10^3^/µL with a median SII value of 1165.11 x10^3^/µL. After one week of treatment, the median platelet count was 289.5 x10^3^/µL (±136.5), the absolute neutrophil count was 8045 x10^3^/µL, the absolute lymphocyte count was 950 x10^3^/µL, and the median SII value was 2711.82 x10^3^/µL (Table 2).

**Table 1:** Characteristics of patients included in the study

Variables		Frequency	Percentage
Age Category	≥60 years	127	41.0%
	<60 years	183	59.0%
Sex	Male	179	57.7%
	Female	131	42.3%
Patient Condition	Death	94	30.3%
	Survived	216	69.7%
Severity of Illness	Mild-Moderate	121	39%
	Severe Clinical	189	61%
Diabetes Mellitus	Yes	125	40.3%
	No	185	59.7%
Hypertension	Yes	151	48.7%
	No	159	51.3%
Chronic Kidney Disease	Yes	40	12.9%
	No	270	87.1%
Heart Failure	Yes	48	15.5%
	No	262	84.5%
BMI Category	≥25 kg/m^2^	134	44.2%
	<25 kg/m^2^	169	55.8%
Additional Therapy of COVID-19	Yes	130	41.9%
	No	180	58.1%

**Table 2:** Blood examination at admission and one week of hospital admission SII: systemic immune-inflammation index

Variable		Median	Minimum	Maximum
At admission	Platelet Count (x10^3^/µL)	211.50	22	733
	Absolute Neutrophil Count (x10^3^/µL)	5890	970	58697
	Absolute Lymphocyte Count (x10^3^/µL)	995	220	11240
	SII (x10^3^/µL)	1165.11	38.65	22831.22
After one week of hospital admission	Platelet Count (x10^3^/µL)	289.50	11	748
	Absolute Neutrophil Count (x10^3^/µL)	8045	300	55614
	Absolute Lymphocyte Count (x10^3^/µL)	950	160	25660
	SII (x10^3^/µL)	2711.82	2.87	23490.31

**Figure 2:**
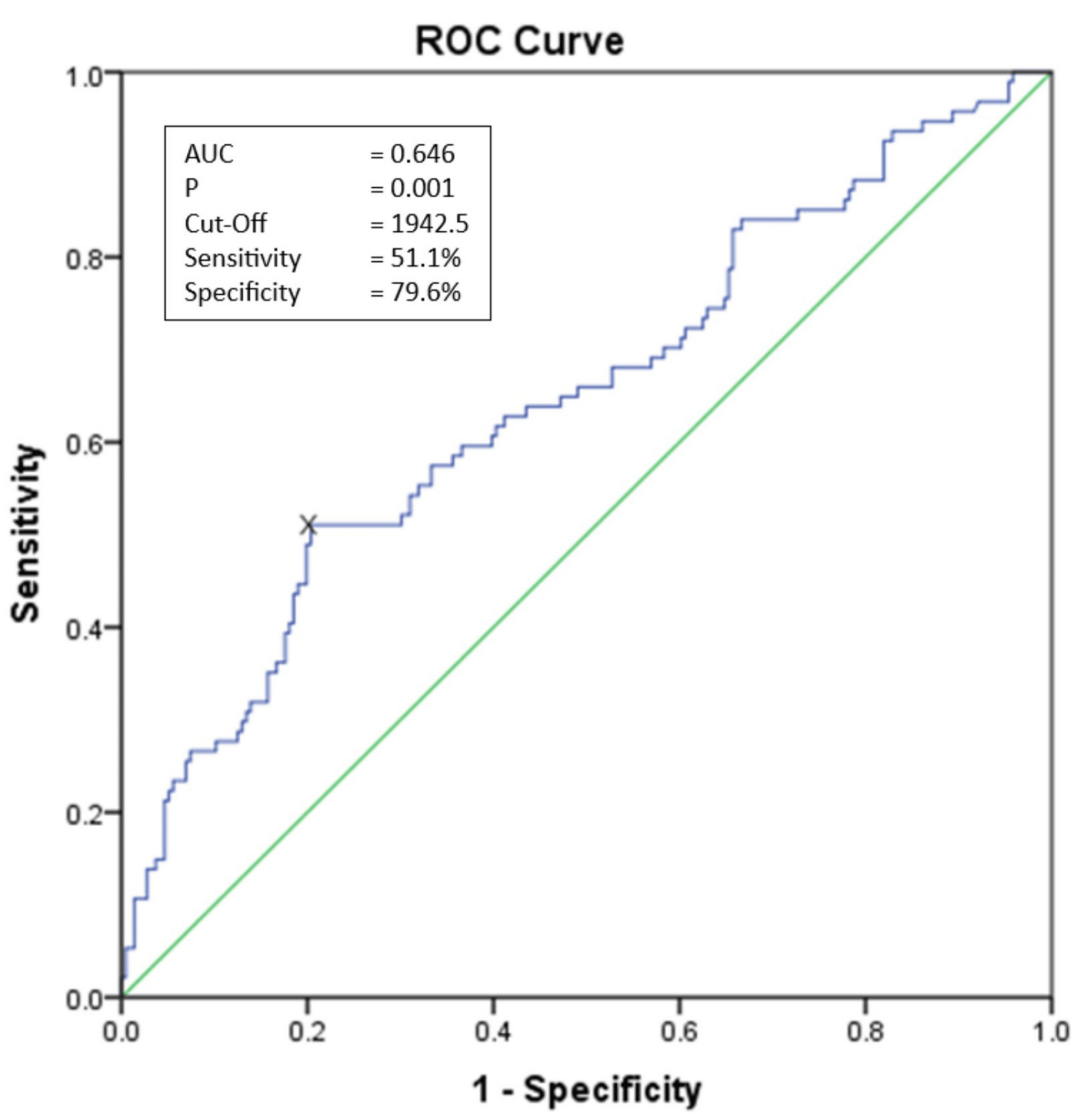
The ROC value of the SII at admission with AUC of 0.646 and p=0.001. The optimal SII cut-off value of 1942.5x103/µL was obtained with 51.1% sensitivity and 79.6% specificity in predicting COVID-19 mortality. AUC: area under the curve; ROC: receiver operating characteristic; SII: systemic immune-inflammation index

Figure 2 shows the ROC value of the SII at admission with an area under the curve (AUC) of 0.646 and p=0.001. The optimal SII cut-off value of 1942.5x10^3^/µL was obtained with 51.1% sensitivity and 79.6% specificity in predicting COVID-19 mortality. Bivariate analysis is shown in Tables 3, 4. In the persistence of high SII (>1942.5x10^3^/µL), 57.3% of patients died and 42.7% survived; the persistence of high SII was associated with the risk of death in patients with COVID-19 with an OR of 5.17 (95% CI: 3.00-8.89). Other variables associated with COVID-19 mortality were the severity of COVID-19 (p=0.000), having diabetes mellitus (p=0.003), chronic kidney disease (p=0.033), and receiving specific additional therapy for COVID-19 (p=0.000). Multivariate analysis showed that the persistence of high SII was independently associated with chronic kidney disease (p= 0.043), COVID-19 severity (p= 0.012), and receiving specific additional therapy for COVID-19 (p=0.004) were independently associated with COVID-19 mortality (p <0.05) (Table 5).

**Table 3:** Bivariate analysis of variables and patient status SII: systemic immune-inflammation ^* ^significant p<0.05, Log Regression

Variables		Death		Survived		P value	OR	95% CI
		N	%	N	%			
Age Category	≥60 years	44	34.6%	83	65.4%	0.168	1.41	0.86-2.30
	<60 years	50	27.3%	133	72.7%			
Sex	Male	60	33.5%	119	66.5%	0.153	1.43	0.87-2.36
	Female	34	26.0%	97	74.0%			
BMI Category	≥25 kg/m^2^	41	30.6%	93	69.4%	0.927	1.02	0.62-1.66
	<25 kg/m^2^	50	29.6%	119	70.4%			
Severity of Illness	Mild-Moderate	17	14%	104	86%	0.000*	4.20	2.33-7.58
	Severe Critical	77	40.7%	112	59.3%			
Diabetes Mellitus	Yes	50	40.0%	75	60.0%	0.003*	2.13	1.30-3.49
	No	44	23.8%	141	76.2%			
Hypertension	Yes	53	35.1%	98	64.9%	0.075	1.55	0.95-2.53
	No	41	25.8%	118	74.2%			
Chronic Kidney Disease	Yes	18	45.0%	22	55.0%	0.033*	2.08	1.06-4.11
	No	76	28.1%	194	71.9%			
Heart Failure	Yes	19	39.6%	29	60.4%	0.131	1.63	0.86-3.09
	No	75	28.6%	187	71.4%			
Additional Therapy	Yes	66	50.8%	64	49.2%	0.000*	5.59	3.26-9.50
	No	28	15.6%	152	84.4%			
High Persistence SII (>1942.5 x10^3^)	Yes	47	57.3%	35	42.7%	0.000*	5.17	3.00-8.89
	No	47	20.6%	181	79.4%			

**Table 4:** Bivariate analysis of variables and SII persistence SII: systemic immune-inflammation index ^*^ significant p<0.05, Log Regression

Variables		High Persistence SII				P value
		Yes		No		
		Frequency	Percentage	Frequency	Percentage	
Age Category	≥60 years	39	47.6%	88	38.6%	0.190
	<60 years	43	52.4%	140	61.4%	
Sex	Male	58	70.7%	121	53.1%	0.006*
	Female	24	29.3%	107	46.9%	
BMI Category	≥25 kg/m^2^	33	41.3%	101	45.3%	0.603
	<25 kg/m^2^	47	58.8%	122	54.7%	
Severity of Illness	Mild-Moderate	15	12.4%	106	87.6%	0.000*
	Severe Critical	67	35.4%	122	64.6%	
Diabetes Mellitus	Yes	38	46.3%	87	38.2%	0.237
	No	44	53.7%	141	61.8%	
Hypertension	Yes	38	46.3%	113	49.6%	0.699
	No	44	53.7%	115	50.4%	
Chronic Kidney Disease	Yes	8	9.8%	32	14.0%	0.442
	No	74	90.2%	196	86.0%	
Heart Failure	Yes	13	15.9%	35	15.4%	0.914
	No	69	84.1%	193	84.6%	
Additional Therapy	Yes	82	63.1%	48	36.9%	0.000*
	No	0	0	180	100%	

**Table 5:** Multivariate analysis of variables and patient status SII: systemic immune-inflammation index ^* ^significant p<0.05

Variables	p value	OR	95.0% CI	
			Lower	Upper
Diabetes Mellitus	0.072	1.664	0.995	2.900
Chronic Kidney Disease	0.043*	2.206	1.027	4.741
High Persistence of SII	0.034*	2.283	1.066	4.887
Additional Therapy	0.012*	2.609	1.237	5.503
Severity of Illness	0.004*	2.597	1.368	4.931

## Discussion

The majority of subjects in this study were aged <60 years, with a male predominance. This is in line with a study conducted in China, which found a mean age of 61.2 ± 0.86, but with a female predominance [9]. Consistent with this earlier study, we found no significant association between age group or gender and mortality.

Poor health status, declining organ function, various comorbidities, and lack of personal hygiene in the elderly patient have contributed to an increased severity of COVID-19. Elderly patients who are infected or suspected with SARS-CoV-2 have a higher risk of experiencing cytokine storms due to declining immunity related to immunosenescence, which increases the risk of mortality. The management of COVID-19 in the elderly population must consider the adverse effects of therapy, especially the treatment for cytokine storms and the administration of corticosteroids [1,11].

Most subjects in earlier studies were in the BMI category of >25 kg/m^2^. Patients with obesity have a three times higher risk of hospitalization for COVID-19 than those with a normal BMI. Obese and overweight subjects are also more prone to experiencing severe and critical COVID-19. This is exacerbated by reduced lung capacity and ventilation, thereby increasing mortality and the need for ventilators [12,13].

Among the study subjects, 48.7% had hypertension, 40.3% had diabetes, and a minority had heart failure or coronary artery disease. These clinical characteristics are consistent with several previous studies that found hypertension to be the most common comorbidity in COVID-19, followed by diabetes mellitus, heart failure, and chronic kidney disease. These conditions increase mortality in COVID-19 [14-16].

Patients with hypertension express more angiotensin-converting enzyme 2 (ACE2) receptors than normal subjects, which are necessary for viral entry into host cells. Thus, consuming ACE inhibitors and angiotensin receptor blockers (ARBs), which are a class of drugs often used in hypertension therapy, may increase the number of ACE2 receptors, potentially increasing the binding of SARS-CoV-2 in the lungs. However, experimental studies have shown that ACE2 has a protective effect against lung tissue damage. ACE2 converts angiotensin II to angiotensin 1-7, reducing the inflammatory effect of angiotensin II and increasing the anti-inflammatory potential of angiotensin 1-7. These two anti-hypertensive drugs may play a role in reducing systemic inflammation, particularly in the lungs, heart, and kidneys, as well as reducing the risk of worsening ARDS, myocarditis, and acute kidney injury [1]. Reports on the risk of severe complications in patients with hypertension may be complicated by age or other comorbidities. To date, hypertension has not been proven to be an independent risk factor for severe complications or mortality in COVID-19 [1].

We discovered a significant association between diabetes mellitus and COVID-19 mortality through the bivariate analysis, although this association was inconsistent in the multivariate analysis. This outcome is comparable to many previous studies that found that diabetes mellitus can increase the risk of death in COVID-19 patients. Dysregulation of glucose metabolism, insulin resistance, immunomodulation, and increased activity of the renin angiotensin aldosterone system (RAAS) in diabetes leads to increased inflammation in COVID-19. Disease progression can also be accelerated and worsened by the condition of hyperglycemia [17].

We found that chronic kidney disease is also an independent risk factor for death during hospitalization in COVID-19, with an OR of 2.206 (p=0.043). Patients with chronic kidney disease, especially those on dialysis or receiving kidney transplants, are immunocompromised and are associated with chronic uremia, other co-morbidities, and immunosuppressant therapy, which increases their susceptibility to COVID-19 and associated mortality [1,18].

Patients with cardiovascular comorbidities are at higher risk of developing severe symptoms and complications related to COVID-19. The prevalence of hypertension and cardiovascular disease in the population with COVID-19 is 17.1%, with a hospitalization rate of 16.4%, and they are at two to three times more at risk of experiencing severe COVID. In patients with heart failure, the cardiac burden is increased due to the fluid retention that occurs following endothelial activation during a cytokine storm [1,19,20]. However, in contrast to previous findings, we did not find a significant association between heart failure and COVID-19 mortality (p=0.131).

In this study, the severity of COVID-19 and the administration of additional specific therapy for COVID-19 were shown to be predictors of COVID-19 in-hospital mortality with an OR of 2.597 (CI 1.368-4.931; p=0.004) and 2.609 (CI 1.237-5.503; p=0.012), respectively. Mehta et al. also found similar results, in which patients with severe and critical COVID-19 had a higher mortality rate within the first 14 days of hospitalization [21]. Critical COVID-19 is caused by a hyper-inflammatory condition, namely hypercytokinemia or cytokine storm. Increased cytokines cause endothelial dysfunction, vascular damage, and metabolic dysregulation, which results in damage to various organs. Acute phase-responding cytokines such as tumor necrosis factor (TNF) and interleukin (IL)-1β, as well as chemotactic cytokines such as IL-8 and monocyte chemoattractant protein-1 (MCP-1), are increased early in hypercytokinemia. This facilitates IL-6 binding to its receptors and forming a complex that acts on GP130. This protein regulates the levels of IL-6, MCP-1, and granulocyte-macrophage colony-stimulating factor (GM-CSF) through the Janus kinase-signal transducer and activator of the transcription (JAK-STAT) pathway, which worsens the inflammatory process. Interleukin-6, alongside other pleiotropic cytokines, induces an acute phase response, increasing serum ferritin, complement, C-reactive protein, and pro-coagulant factors, many of which can be measured by laboratory blood tests [22].

We obtained an SII value of 1942.5x10^3^ as the optimal cut-off for predicting COVID-19 mortality with a sensitivity of 51.1% and a specificity of 79.6%. Consistent with these results, in a study by Fois et al. on 119 patients, an SII cut-off value of 1835x10^9^ was reported with a sensitivity of 55% and a specificity of 75% [8]. In another study involving 326 subjects, Li et al. set the SII cut-off at 1293 x10^9^ with sensitivity and specificity of 69.9% and 70.8%, respectively [9].

Out of the 310 subjects, 82 subjects had persistent SII values ​​above the cut-off value both at admission and after one week of hospitalization and were grouped as having high persistent SII. In both bivariate and multivariate analyses, high persistent SII consistently was proven to be a predictor of mortality during hospitalization in COVID-19 patients with an OR of 5.17 and 2.283, respectively. Several previous studies have assessed the relationship between SII at admission and mortality during hospitalization [8,9,23,24]. These studies have shown that high SII at admission is a good predictor of COVID-19 in-hospital mortality. An imbalance in immunological control and systemic inflammation is indicated by the elevated SII value, which functions as a composite signal involving certain hematological cells. A lengthier virus clearance time could result from this imbalance, which is likely to impair the immune system's reaction to viral infections.

A high persistent SII indicates a cytokine storm that occurs within the first one to two weeks of SARS-CoV-2 infection, in which the activation of the innate and adaptive immune systems occurs, accompanied by the release of chemokines and pro-inflammatory cytokines. High serum cytokine levels are directly related to lymphopenia, which may lead to reduced viral clearance by cytotoxic T cells. On the other hand, macrophages and dendritic cells trigger an initial immune response, including lymphocytosis and cytokine release, but this could in lymphocyte destruction in an attempt to limit SARS-CoV-2 infection. Lymphopenia mainly occurs in patients with severe and critical degrees of COVID, in line with the need for intensive care. Cytokine production becomes rapid and irregular, damaging healthy cells, especially in the lungs and other organs, including the kidneys, heart, blood vessels, and brain [19,25-29].

Limitations

This study did not assess the SARS-CoV-2 virus strain that infected, because in the study samples, not all were examined for whole genome sequencing (WGS) or S-gene target failure (SGTF) and or single nucleotide polymorphism (SNP) based on PCR, where this examination was only performed on a few patients as samples. This study did not analyze secondary infections from the subjects studied; this could cause changes in the SII value.

## Conclusions

This study shows a significant association between the persistence of high SII as a prognostic factor for COVID-19 mortality, with a cutoff value of 1942.59x10^9^, which has a sensitivity of 51.1% and a specificity of 79.6%. This study still needs support from further studies due to our limitations. 
